# Association Between Lipid-Lowering Therapy and Differences in the Distribution of LDL-C, apoB and non-HDL-C

**DOI:** 10.3390/jcm15010026

**Published:** 2025-12-20

**Authors:** Marcin Ziółkowski, Karolina Obońska, Jakub Ratajczak, Piotr Adamski, Maciej Banach, Krzysztof Chlebus, Klaudyna Grzelakowska, Piotr Jankowski, Magdalena Krintus, Jacek Kryś, Ewa Laskowska, Natalia Mrzywka, Piotr Niezgoda, Małgorzata Ostrowska, Przemysław Podhajski, Grzegorz Skonieczny, Bożena Sosnowska, Łukasz Szarpak, Małgorzata Topolska, Julia Umińska, Alicja Rzepka-Cholasińska, Eliano Pio Navarese, Jacek Kubica

**Affiliations:** 1 Antoni Jurasz University Hospital No. 1 in Bydgoszcz, 85-094 Bydgoszcz, Poland; m.ziolkowski@jurasza.pl (M.Z.); jacek.krys@gmail.com (J.K.); 2 Ludwik Rydygier Collegium Medicum in Bydgoszcz, Nicolaus Copernicus University in Torun, 85-094 Bydgoszcz, Poland; jakub.ratajczak@cm.umk.pl (J.R.); piotr.adamski@cm.umk.pl (P.A.); klaudyna.grzelakowska@gmail.com (K.G.); krintus@cm.umk.pl (M.K.); ewa.laskowska@cm.umk.pl (E.L.); piotr.niezgoda@cm.umk.pl (P.N.); m.ostrowska@cm.umk.pl (M.O.); przemyslaw.podhajski@cm.umk.pl (P.P.); julia.uminska@cm.umk.pl (J.U.); alicja.rzepka@cm.umk.pl (A.R.-C.); elianonavarese@gmail.com (E.P.N.); jkubica@cm.umk.pl (J.K.); 3 Faculty of Medicine, The John Paul II Catholic University of Lublin (KUL), 20-950 Lublin, Poland; maciej.banach@icloud.com; 4 Department of Preventive Cardiology and Lipidology, Medical University of Lodz (MUL), 90-419 Lodz, Poland; bozena.sosnowska@umed.lodz.pl; 5 Ciccarone Center for the Prevention of Cardiovascular Disease, Johns Hopkins University School of Medicine, Baltimore, MD 21218, USA; 6 Liverpool Centre for Cardiovascular Science (LCCS), Liverpool L7 8TX, UK; 7 First Department of Cardiology, Medical University of Gdansk, 80-210 Gdansk, Poland; krzysztof.chlebus@gumed.edu.pl; 8 Department of Internal Medicine and Geriatric Cardiology, Medical Centre of Postgraduate Education, 01-813 Warsaw, Poland; piotrjankowski@interia.pl; 9 Department of Epidemiology and Health Promotion, School of Public Health, Centre of Postgraduate Medical Education, 01-826 Warsaw, Poland; 10 American Heart of Poland, 64-920 Pila, Poland; natalia.mrzywka@onet.eu; 11 Department of Cardiology and Intensive Cardiac Care Unit, District Polyclinic Hospital, 87-100 Torun, Poland; grzegorz.skonieczny@onet.eu; 12 Institute of Medical Science, Collegium Medicum, The John Paul II Catholic University of Lublin (KUL), 20-950 Lublin, Poland; lukasz.szarpak@gmail.com; 13 Department of Clinical Research and Development, LUXMED Group, 02-678 Warsaw, Poland; 14 Henry JN Taub Department of Emergency Medicine, Baylor College of Medicine, Houston, TX 77030, USA; 15 American Heart of Poland, 40-028 Katowice, Poland; malgorzata.topolska@ahop.pl

**Keywords:** ApoB, dyslipidemia, LDL, lipid-lowering therapy, non-HDL-C

## Abstract

**Background:** The diagnosis of hypercholesterolemia relies on the laboratory assessment of lipid parameters. This study aimed to evaluate differences in the distribution of low-density lipoprotein cholesterol (LDL-C), non-high-density lipoprotein cholesterol (non-HDL-C), and apolipoprotein B (apoB) concentrations according to the presence and type of lipid-lowering therapy (LLT). **Methods:** This retrospective analysis included consecutive patients who had at least one measurement of LDL-C, apoB, and non-HDL-C between March and November 2024 in a high-volume tertiary hospital. All lipid fractions were expressed as the percentages of measurements above or below cut-off values established by the recent ESC guidelines. Subgroup analysis based on LLT type was performed, with patients categorized as receiving either single or combined LLT. **Results:** A total of 5048 patients were included in the analysis. Among patients receiving LLT, most were on statin monotherapy (77.3%), predominantly atorvastatin. Combined therapy, primarily statin plus ezetimibe, was used in 22.7% of treated patients. Discordance between on-target apoB levels and elevated LDL-C concentrations occurred in 26.6% of untreated and 13.6% of all treated patients, and in 15.1% and 8.6% of single and combined-LLT patients, respectively. Similarly, discordance between on-target non-HDL-C and elevated LDL-C levels was observed in 13.5% of untreated and 7.5% of all treated patients, and in 8.4% and 4.8% of single and combined-LLT patients, respectively. **Conclusions:** Classification of hyperlipidemia based on LDL-C, non-HDL-C, and apoB concentrations reveals significant discrepancies between these markers, especially between LDL-C and apoB. LLT reduces these discrepancies with combined LLT being particularly effective.

## 1. Introduction

Hypercholesterolemia remains a major risk factor for atherosclerotic cardiovascular disease (ASCVD) [1]. The diagnosis of hypercholesterolemia is primarily based on low-density lipoprotein cholesterol (LDL-C) concentration measurements [2]. However, in specific patient populations—those with elevated triglyceride (TG), diabetes mellitus (DM), obesity, or very low LDL-C concentrations—the assessment of non-high-density lipoprotein cholesterol (non-HDL-C) or apolipoprotein B (apoB) levels is recommended [2]. ApoB can serve as an alternative to LDL-C as the primary measurement for screening, diagnosis, and management, and may be preferred over non-HDL-C, particularly in the aforementioned cases [2].

LDL-C, apoB, and non-HDL-C provide related but distinct information about lipid status. LDL particles consist of both lipids and proteins, with an apolar core primarily composed of cholesteryl esters, small amounts of TG, and a small amount of free unesterified cholesterol. This core is surrounded by an outer shell consisting of a phospholipid monolayer containing the majority of the free unesterified cholesterol molecules and a single copy of apoB-100 [3]. LDL particles are highly heterogeneous, particularly regarding the chemical composition of their core lipids [3]. Variations in these proportions create differences in the average mass of cholesterol per apoB molecule [4]. The assessment of LDL-C, apoB, and non-HDL-C provides complementary information due to their different compositions. ApoB-100 is present in multiple lipoprotein particles: LDL, very-low-density lipoprotein (VLDL), intermediate-density lipoprotein (IDL), and lipoprotein(a) [Lp(a)] [2,4,5]. In contrast, non-HDL-C reflects the plasma concentration of cholesterol in all apoB-containing lipoproteins, including LDL, VLDL, IDL, chylomicrons (CM), CM remnants, VLDL remnants, and Lp(a) [5]. The concentration of lipid parameters is associated with various determinants, and the impact of lipid-lowering therapy (LLT) is crucial in this aspect [6,7].

The aim of this study was to evaluate differences in the distribution of LDL-C, non-HDL-C, and apoB concentrations according to the presence and type of lipid-lowering therapy (LLT).

## 2. Materials and Methods

**Figure 1:**
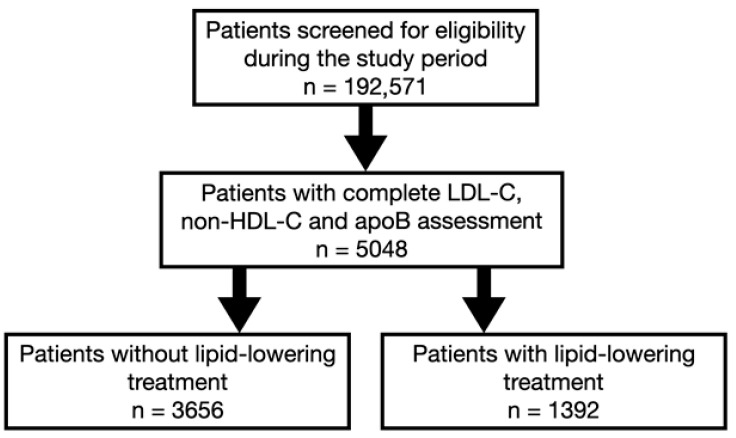
Study flowchart of patients.

This analysis is part of the Jurasz Lipid Study (JLS), a retrospective, single-center registry that includes data from consecutive adult patients who underwent at least one lipid profile assessment between March and November 2024 at the Antoni Jurasz University Hospital No. 1 in Bydgoszcz, Poland. For patients with multiple lipid profile measurements during the analyzed period, the study used the first measurement that included complete lipid parameters [total cholesterol (TC), LDL-C, HDL-C, non-HDL-C, TG, apoB, and Lp(a)] for analysis. During the study period, 192,571 patients were screened for eligibility (Figure 1). We reviewed digital medical records using information recorded in the medical interview forms and diagnostic codes according to the International Classification of Diseases (ICD-10 and ICD-9) (Table 1).

Patient characteristics included age, gender, height, weight, body mass index (BMI), comorbidities, smoking status, systolic blood pressure, and LLT. If applicable, the SCORE2 (Systematic Coronary Risk Evaluation 2) and SCORE2-OP (Systematic Coronary Risk Estimation 2—Older Persons) scales were used to assess cardiovascular (CV) risk for all patients with sufficient data [9]. Since Poland is classified as a high-risk region, the appropriate scale was used for CV risk assessment [9].

Biochemical parameters assessed in the JLS included estimated glomerular filtration rate (eGFR) and comprehensive lipid profile, including TC, LDL-C (measured directly), HDL-C, non-HDL-C, TG, apoB, and Lp(a). The laboratory tests were conducted in both fasting and non-fasting conditions. The Alinity platform (Abbott Laboratories, Chicago, IL, USA) was used to perform the measurements. ApoB was measured on the Abbott Alinity c platform using an immunoturbidimetric method. The assay demonstrates analytical precision with coefficients of variation of 2.6–3.7% across control levels (43–123 mg/dL).

Among patients with measured LDL-C, non-HDL-C, and apoB, the differences in the distribution of these lipid fractions were assessed. To compare the distribution, all fractions were expressed as a percentage of measurements above and below cut-off values defined according to the recent guidelines: 100 mg/dL for LDL-C, 130 mg/dL for non-HDL-C, and 100 mg/dL for apoB [9]. The cut-off points for moderate cardiovascular risk were selected considering that all subsequent patients hospitalized and consulted in outpatient clinics during the analyzed period would be included in the study, regardless of their burden. Discordances were defined as follows: for LDL-C and non-HDL-C: LDL-C ≤ 100 mg/dL and non-HDL-C > 130 mg/dL and LDL-C > 100 mg/dL and non-HDL-C ≤ 130 mg/dL; for apoB and LDL-C as: LDL-C ≤ 100 mg/dL and apoB > 100 mg/dL and LDL-C > 100 mg/dL and apoB ≤ 100 mg/dL; for non-HDL-C and apoB as: non-HDL-C ≤ 130 mg/dL and apoB > 100 mg/dL and non-HDL-C > 130 mg/dL and apoB ≤ 100 mg/dL.

The subgroup analysis was performed based on the type of LLT. Patients were divided into two groups according to whether they received single or combined LLT.

The study received approval of the Ethics Committee of The Nicolaus Copernicus University in Torun, Collegium Medicum in Bydgoszcz (KB 49/2025) and was conducted in accordance with the Declaration of Helsinki and Good Clinical Practice principles.

Statistical analysis was performed using the IBM SPSS Statistics software version 29 (IBM Corporation, Armonk, NY, USA). Continuous variables were presented as medians with interquartile range (IQR). The distribution of data was assessed using the Shapiro-Wilk test and histogram analysis. Differences in data distribution between continuous variables were assessed with the Mann-Whitney U or the Student *t*-test for two variables and the Kruskal-Wallis test for more than two variables. Categorical variables were presented as counts with percentages. The chi-squared test was used to compare differences between categorical variables. For statistical significance a two-sided *p*-value < 0.05 was used. Logistic regression analysis was performed to determine the independent variables associated with discordant LDL-C/apoB, LDL-C/non-HDL-C, and apoB/non-HDL-C. Initially, univariate regression analysis was conducted. Variables with *p*-value < 0.1 were further analyzed in the multivariate logistic regression models.

## 3. Results

Overall, 5048 patients had complete LDL-C, non-HDL-C, and apoB measurements performed during the analyzed period and were included in the study. In general, patients receiving LLT had a significantly higher CV disease burden and less favorable metabolic profile than those without LLT (Table 2).

Most patients on LLT received statin monotherapy (77.3%), predominantly atorvastatin. Combined therapy, mostly comprising statin plus ezetimibe, was administered to 22.7% of treated patients. There were no patients treated with PCSK9 inhibitors or siRNA in the analyzed period. Detailed data on the LLT used are presented in Table 3.

In the study group, the cut-off values for LDL-C, non-HDL-C and apoB were exceeded in 45.7%, 35.1%, and 23.2% of participants, respectively.

**Figure 2:**
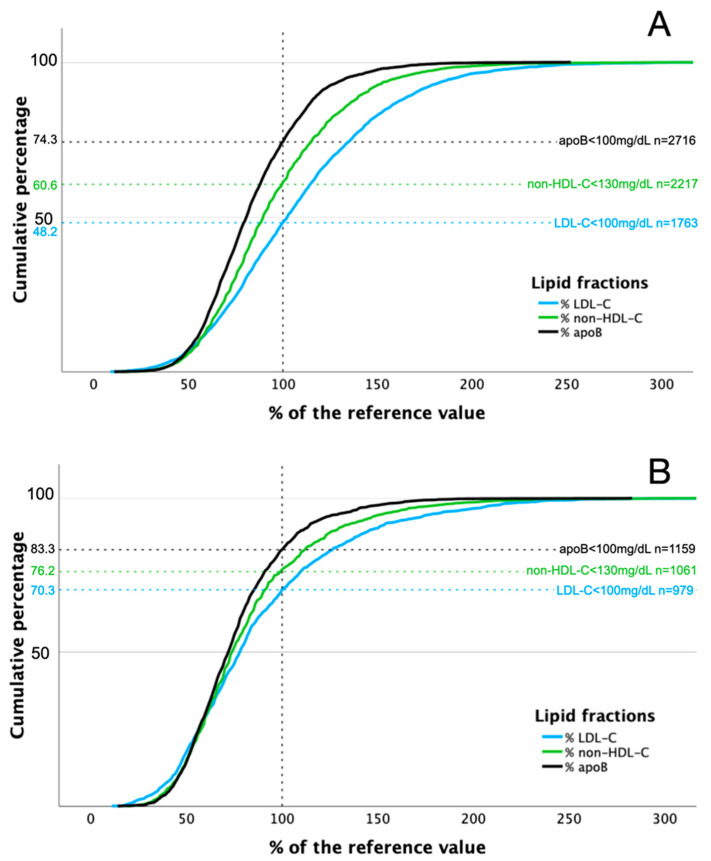
Distribution of LDL-C, non-HDL-C, and apoB concentrations in relation to reference values in patients without (**A**) and with (**B**) lipid-lowering treatment.

**Figure 3:**
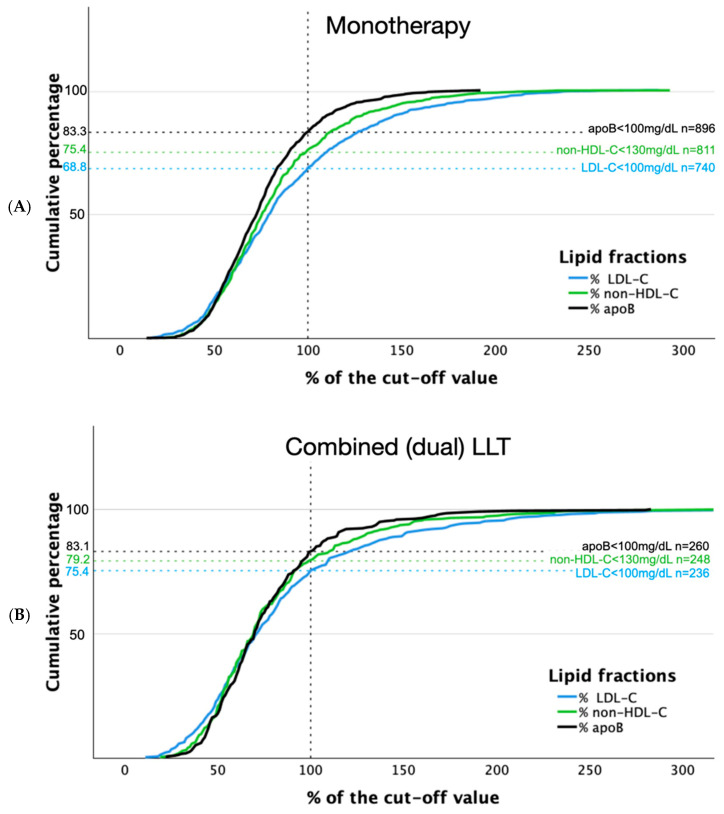
Distribution of LDL-C, non-HDL-C, and apoB concentrations in relation to reference values in patients with lipid-lowering monotherapy (**A**) and combined lipid-lowering treatment (**B**).

Distributions of LDL-C, non-HDL-C, and apoB in relation to reference values in patients with and without LLT are shown in Figure 2. Concentrations of analyzed lipid fractions were below threshold more often in patients receiving combined LLT compared to those on monotherapy: 75.4% vs. 68.8% and 79.2% vs. 75.4%, for LDL-C and non-HDL-C, respectively (Figure 3).

Similar rates were observed below the cut-off value in patients on monotherapy and combined therapy for apoB (83.3% vs. 83.1%, respectively).

The frequencies of discordance between apoB/LDL-C, apoB/non-HDL-C and LDL-C/non-HDL-C in the study population according to the LLT used and its type are presented in Table 4. The results of univariate regression analysis for variables associated with discordant apoB/LDL-C, apoB/non-HDL-C, and LDL-C/non-HDL-C are presented in the Supplementary Materials (Tables S1–S3). In the multivariate regression analysis, the discordance between LDL-C/apoB was independently associated with the use of LLT [OR 0.70 (95% CI 0.56–0.86), *p* < 0.001], age [OR 0.986 (95% CI 0.983–0.99), *p* < 0.001], male gender [OR 0.79 (95% CI 0.67–0.92), *p* = 0.003], DM [OR 0.60 (95% CI 0.47–0.76), *p* < 0.001], and mitral valve disease [OR 0.73 (95% CI 0.57–0.94), *p* = 0.015]. Older age [OR 0.98 (95% CI 0.978–0.986), *p* < 0.001] and DM [OR 0.67 (95% CI 0.49–0.91), *p* = 0.01] were associated with lower LDL/non-HDL-C discordance. The discordant apoB/non-HDL-C were independently associated with LLT [OR 0.68 (95% CI 0.52–0.90), *p* = 0.007], DM [OR 0.61 (95% CI 0.44–0.83), *p* = 0.002] and ASCVD [OR 0.78 (95% CI 0.63–0.88), *p* = 0.029] in the multivariate model.

**Figure 4:**
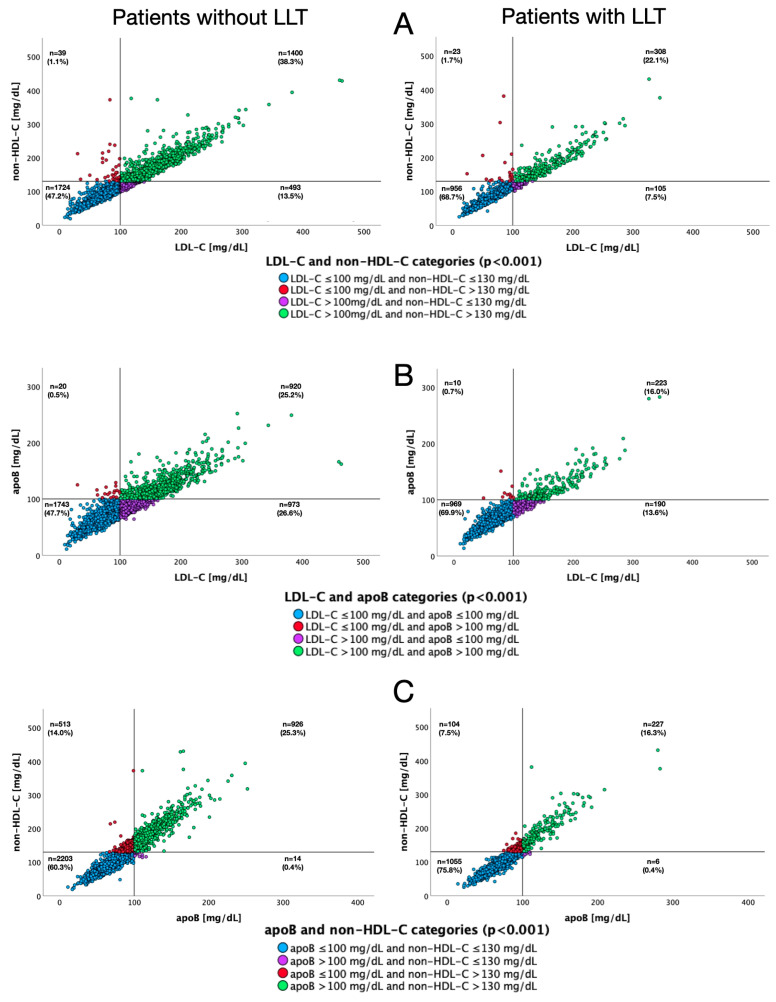
Scatter plots for pairs of lipid fractions regarding their target values, depending on the use of lipid-lowering therapy. LDL-C and non-HDL-C (**A**), apoB and LDL-C (**B**), non-HDL-C and apoB (**C**).

**Figure 5:**
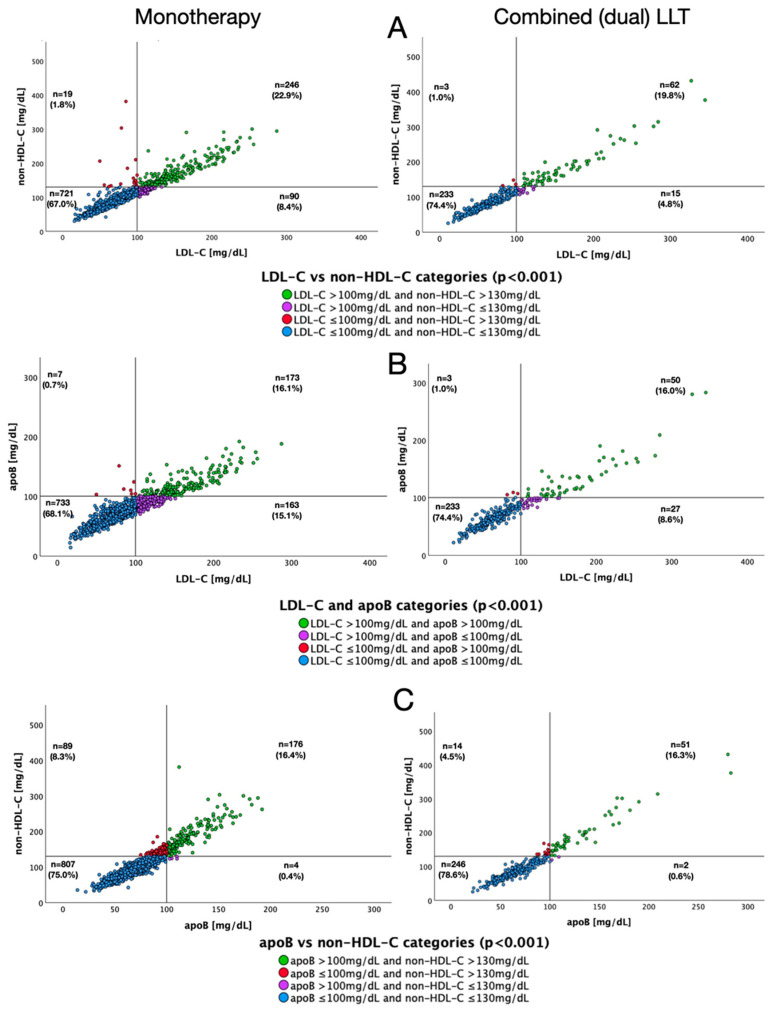
Scatter plots for pairs of lipid fractions regarding their target values, depending on the type of lipid-lowering therapy. LDL-C and non-HDL-C (**A**), apoB and LDL-C (**B**), non-HDL-C and apoB (**C**).

Figure 4 depicts scatter plots for different pairs of lipid fractions relative to target values for each of them, both in patients with and without LLT. Figure 5 presents analogous data according to the type of LLT used. Concomitant non-HDL-C concentrations below target value and elevated LDL-C levels were observed in 13.5%, 7.5%, 8.4% and 4.8% of patients not receiving LLT, receiving any LLT, single LLT and combined LLT, respectively. Discordance between target apoB levels and increased LDL-C concentrations occurred in 26.6%, 13.6%, 15.1% and 8.6% of patients not receiving LLT, receiving any LLT, single LLT or combined LLT, respectively.

## 4. Discussion

In a large consecutive patient cohort spanning both inpatient and outpatient settings, the Jurasz Lipid Study (JLS) demonstrated significant treatment-dependent variations in the distribution of atherogenic lipid markers (LDL-C, non-HDL-C, and apoB).

The proportion of untreated patients with LDL-C, non-HDL-C, and apoB levels exceeding the target cut-off points for moderate CV risk were 51.8%, 39.4%, and 25.7%, respectively, compared to 29.7%, 23.8%, and 16.7% in those receiving LLT. Notably, many patients receiving LLT failed to achieve even the target values for moderate CV risk, despite being categorized as high or very high risk. The NATPOL 2011 study reported higher percentages of patients exceeding cut-off values for moderate cardiovascular risk: 74.91%, 63.7%, and 30.91% for LDL-C, non-HDL-C, and apoB, respectively [10]. Consequently, the concordance/discordance between LDL-C, non-HDL-C, and apoB concentrations varied substantially between the NATPOL 2011 study [10] and the JLS. Interpreting these inter-study discrepancies presents significant challenges. One possible explanation lies in the demographic differences between the studied populations, the NATPOL 2011 participants were younger than the JLS cohort and exhibited higher lipid fraction concentrations [11].

Other studies have yielded inconsistent results. The Multi-Ethnic Study of Atherosclerosis (MESA) [12] found that among patients without ASCVD not receiving LLT, discordant low apoB/high LDL-C and high apoB/low LDL-C patterns occurred in 9% and 8.4% of patients, respectively. In a middle-aged Korean male population (*n* = 14,205), these discordances were observed in 7.8% and 5.3% of subjects, respectively [13].

In the Copenhagen General Population Study [14], which included 13,015 patients treated with statins, the rate of discordance was 13% for both comparisons. In the same cohort, low non-HDL-C/high LDL-C and high non-HDL-C/low LDL-C patterns were reported in 7% and 8% of patients, respectively [14]. The National Health and Nutrition Examination Survey 2009–2010 in the United States [15] found that discordance of high apoB/low LDL-C and low apoB/high LDL-C each occurred in 7.8% of participants, while 6.3% had discordantly high non-HDL-C with low LDL-C or low non-HDL-C with high LDL-C [15]. It should be noted that several of these studies used medians rather than cut-off points, potentially affecting the reported proportions of discordance. In a meta-analysis of 38,153 patients treated with statins, Boekholdt et al. [16] demonstrated discordance rates of 3.76% for high non-HDL-C/low LDL-C and 7.53% for low non-HDL-C/high LDL-C.

The JLS presents the distribution of apoB, LDL-C, and non-HDL-C in a large group of patients with and without LLT. The differences between distributions of the analyzed lipid parameter pairs are complex. Combined LLT is associated with a significant reduction in discordance between compared pairs of lipid fractions, with the largest remaining discrepancy being low apoB/high LDL-C at 8.6%. This reduction may result from fewer atherogenic particles following intensive LLT, as well as the differential effects of therapy on various lipid fractions. The independent nature of the untreated and treated groups in the JLS may influence the analysis results. A detailed assessment of lipid fraction distributions before and after LLT initiation would help resolve this issue.

### Strengths and Limitations

The JLS presents the distribution of LDL-C, non-HDL-C, and apoB in a large cohort of all-comers treated in a multi-specialist hospital. An important strength of this study is its nature of including all consecutive individuals, providing real-life data across a wide spectrum of patients. Nevertheless, the study has several limitations. The main limitation is the study protocol itself, which involves the assessment of lipid fractions at single time points (without regard to food intake), preventing evaluation of the actual impact of therapy on the analyzed parameters. When interpreting the above data, it should be considered that the analyzed groups differed in terms of age, gender and comorbidity burden, which was higher in the group receiving LLT. The cut-off points for moderate CV risk used in the JLS do not reflect the actual treatment goals for patients with higher CV risk. Thus, the actual disparities could potentially be larger. However, the purpose of this paper was to highlight a commonly observed phenomenon. Comparison with other studies is challenging due to varying cut-off points and different statistical approaches applied. Due to the observational nature of the study, LLT varied in both types of medications and dosages. The duration of LLT and adherence to it remain unknown.

## 5. Conclusions

The Jurasz Lipid Study identified a substantial treatment gap in patients with elevated atherogenic lipoprotein levels. Analysis of lipid parameters revealed significant discordance between markers, with the greatest disparities observed between LDL-C and apoB measurements, and more modest differences seen between non-HDL-C and apoB. These discrepancies varied according to LLT patterns and were smallest in patients receiving combined therapy. The use of LLT, age, gender, history of DM, ASCVD, and mitral valve disease were variables independently associated with the discordance between analyzed lipid parameters. These findings underscore the importance of comprehensive lipid assessment and optimized therapeutic strategies in the management of dyslipidemia.

## Figures and Tables

**Table 1:** List of comorbidities and their corresponding ICD-10 and ICD-9 codes. Reprinted from [8].

Comorbidities	ICD-10 and ICD-9
Acute coronary syndromes	I20; I21; I22; I24
Chronic coronary syndromes	I25
CABG/PCI	Z95.1; Z95.5; 36.091; 36.11–16
Aortic stenosis and mitral valve diseases	I34; I35.0; I35.2
Stroke	I61; I62; I63; I64; I69
Other atherosclerotic diseases	I65; I66.; I70; 39.501
Atrial fibrillation	I48
Arterial hypertension	I10; I12; I13
Diabetes mellitus type 1	E10
Diabetes mellitus type 2	E11
Acute/chronic pancreatitis	K85; K86.1
Aortic aneurysm	I71

**Table 2:** Patient characteristics.

Analyzed Parameter		All Patients (*n* = 5 048)	Without LLT (*n* = 3 656)	LLT (*n* = 1 392)	*p*-Value
Age, median (IQR), [y]		64 (45–74)	59 (37–72)	70 (62–77)	<0.001
Male gender, *n* (%)		2599 (51.5)	1800 (49.2)	799 (57.4)	<0.001
BMI, median (IQR), [kg/m^2^]		26.6 (23.0–30.6) *	26.1 (22.5–30.4)	27.5 (24.3–31.4)	<0.001
History of ACS, *n* (%)		874 (17.3)	386 (10.6)	488 (35.1)	<0.001
History of CCS, *n* (%)		1246 (24.7)	622 (17.0)	624 (44.8)	<0.001
History of PCI/CABG, *n* (%)		817 (16.2)	375 (10.3)	442 (31.8)	<0.001
DM, *n* (%)		975 (19.3)	495 (13.5)	480 (34.5)	<0.001
Arterial hypertension, *n* (%)		1988 (39.4)	1098 (30.0)	890 (63.9)	<0.001
SBP, median (IQR), [mmHg]		134 (120–150) *	131 (118.5–150)	139 (122–156)	<0.001
DBP, median (IQR), [mmHg]		80 (70–88) *	80 (70–88)	79 (70–88)	0.304
eGFR, median (IQR), [ml/min]		85.1 (61–101.8) *	89.5 (68.2–108)	70 (47.1–88)	<0.001
Stroke, *n* (%)		872 (17.3)	470 (12.9)	402 (28.9)	<0.001
Peripheral and carotid artery disease, *n* (%)		729 (14.4)	362 (9.9)	367 (26.4)	<0.001
Aortic aneurysm, *n* (%)		126 (2.5)	66 (1.8)	60 (4.3)	<0.001
Aortic stenosis, *n* (%)		402 (8.0)	223 (6.1)	179 (12.9)	<0.001
Mitral valve disease, *n* (%)		730 (14.5)	369 (10.1)	361 (25.9)	<0.001
Atrial fibrillation, *n* (%)		1159 (23.0)	639 (17.5)	520 (37.4)	<0.001
History of pancreatitis, *n* (%)		63 (1.2)	49 (1.3)	14 (1.0)	0.339
Smoker, *n* (%)		692 (13.7)	462 (12.6)	230 (16.5)	<0.001
TC, median (IQR), [mg/dL]		159 (131–196)	167 (137–202)	143 (116–176)	<0.001
LDL-C, median (IQR), [mg/dL]		96 (69–130)	103 (76–136)	78 (57–109)	<0.001
Non-HDL-C, median (IQR), [mg/dL]		112 (88–146)	117.5 (93–152)	97 (76–127)	<0.001
apoB, median (IQR), [mg/dL]		78 (63–99)	81 (66–101)	72 (57–90)	<0.001
HDL-C, median (IQR), [mg/dL]		45 (36–55)	46 (37–56)	42 (33–52)	<0.001
TG, median (IQR), [mg/dL]		97 (69–137)	96 (68–134)	101 (73.3–141.8)	<0.001
Lp(a), median (IQR), [mg/dL]		11 (5–29)	10 (5–26)	14 (6–40)	<0.001
SCORE2/SCORE2-OP categories, *n* (%)	Low-to-moderate	212 (16.8) *	189 (18.5)	23 (9.4)	<0.001
	High	406 (32.1) *	353 (34.6)	53 (21.7)	
	Very high	646 (51.1) *	478 (46.9)	168 (68.9)	

Apo B—apolipoprotein B, ACS—acute coronary syndrome, BMI—body mass index, CABG—coronary artery bypass grafting, CCS—chronic coronary syndrome, DBP—diastolic blood pressure, eGFR—estimated glomerular filtration rate, DM—diabetes mellitus, HDL-C—high-density lipoprotein cholesterol, IQR—interquartile range, LDL-C—low-density lipoprotein cholesterol, Lp(a)—lipoprotein(a), non-HDL-C—non-high-density lipoprotein cholesterol, PCI—percutaneous coronary intervention, TC—total cholesterol, TG—triglycerides, SBP—systolic blood pressure, SCORE2—Systematic Coronary Risk Evaluation 2, SCORE2-OP—Systematic Coronary Risk Estimation 2-Older Persons. SCORE2 and SCORE2-OP were calculated only for suitable patients with required data available. *—parameter was not available for all included patients (BMI *n* = 2727; eGFR *n* = 3517; SBP *n* = 2809; DBP n = 2709; SCORE2/SCORE2-OP *n* = 1264).

**Table 3:** Type of lipid-lowering treatment.

Lipid-Lowering Therapy	*N* (%)
Single LLT	1076 (77.3)
- Statin	1054 (75.7)
Atorvastatin	689 (49.5)
HD (40–80 mg)	210 (15.1)
LD (<40 mg)	416 (29.9)
No info	63 (4.5)
Rosuvastatin	353 (25.4)
HD (20–40 mg)	186 (13.4)
LD (<20 mg)	117 (8.4)
No info	50 (3.6)
Simvastatin	11 (0.8)
20–40 mg	10 (0.7)
No info	1 (0.1)
Pitavastatin	1 (0.1)
- Ezetimibe	17 (1.2)
- Fenofibrate	5 (0.4)
Combined-Dual LLT	313 (22.5)
- Statin + Ezetimibe	308 (22.1)
Atorvastatin/Rosuvastatin HD	206 (14.8)
Other	59 (4.2)
No info	43 (3.1)
- Statin + Fenofibrate	4 (0.3)
Atorvastatin/Rosuvastatin HD	2 (0.1)
Other	2 (0.1)
No info	0 (0.0)
- Ezetimibe + Fenofibrate	1 (0.1)
Combined-Triple LLT	
- Statin + Ezetimibe + Fenofibrate	3 (0.2)
Atorvastatin/Rosuvastatin HD	3

HD—high dose; LD—low dose.

**Table 4:** The incidence of apoB/LDL-C, apoB/non-HDL-C and LDL-C/non-HDL-C discordance in the study population according to the LLT used and its type.

	Total, *n* (%)	Without LLT, *n* (%)	With LLT, *n* (%)	OR [95% CI]	Mono-Therapy, *n* (%)	Combined Therapy, *n* (%)	OR [95% CI]
Discordant apoB/LDL-C	1193 (23.6)	993 (27.2)	200 (14.4)	0.45 [0.38–0.53]	170 (15.8)	30 (9.5)	0.56 [0.37–0.84]
Discordant apoB/non-HDL-C	637 (12.6)	527 (14.4)	110 (7.9)	0.51 [0.41–0.63]	109 (10.1)	19 (6.0)	0.57 [0.34–0.94]
Discordant LDL-C/non-HDL-C	660 (13.0)	532 (14.6)	128 (9.2)	0.60 [0.49–0.73]	92 (8.6)	17 (5.4)	0.60 [0.35–1.02]

## Data Availability

The data presented in this study are available on request from the corresponding author.

## Supplementary Materials

The following supporting information can be downloaded at: https://www.mdpi.com/article/10.3390/jcm15010026/s1, Table S1: Variables associated with discordant LDL-C/apoB based on the univariate and multivar-iate logistic regression analysis. ASCVD—atherosclerotic cardiovascular disease; BMI—body mass index; CI—confidence interval; DM—diabetes mellitus; eGFR—estimated glomerular filtration rate; Lp(a)—lipoprotein (a); OR—odds ratio; Table S2: Variables associated with discordant LDL-C/non-HDL-C based on the univariate and multivariate logistic regression analysis. ASCVD—atherosclerotic cardiovascular disease; BMI—body mass index; CI—confidence interval; DM—diabetes mellitus; eGFR—estimated glomerular filtration rate; Lp(a)—lipoprotein (a); OR—odds ratio; Table S3: Variables associated with discordant apoB/non-HDL-C based on the univariate and multivariate logistic regression analysis. ASCVD—atherosclerotic cardiovascular disease; BMI—body mass index; CI—confidence interval; DM—diabetes mellitus; eGFR—estimated glomerular filtration rate; Lp(a)—lipoprotein(a); OR—odds ratio.

## Abbreviations

The following abbreviations are used in this manuscript:

LDL-C	low-density lipoprotein cholesterol
non-HDL-C	non-high-density lipoprotein cholesterol
apoB	Apolipoprotein B
LLT	lipid-lowering therapy
ASCVD	atherosclerotic cardiovascular disease
TG	triglyceride
DM	diabetes mellitus
VLDL	very-low-density lipoprotein
IDL	intermediate-density lipoprotein
Lp(a)	Lipoprotein (a)
JLS	Jurasz Lipid Study
BMI	body mass index
SCORE2	Systematic Coronary Risk Evaluation 2
SCORE2-OP	Systematic Coronary Risk Estimation 2-Older Persons
CV	cardiovascular
IQR	interquartile range
MESA	The Multi-Ethnic Study of Atherosclerosis
