# Supplementary material for: Association Between Lipid-Lowering Therapy and Differences in the Distribution of LDL-C, apoB and non-HDL-C
Source: J Clin Med. 2025 Dec 20;15(1):26. doi: 10.3390/jcm15010026 (PMC12787146; doi:10.3390/jcm15010026)
Supplement: Supplementary file 1 [file jcm-15-00026-s001.zip › jcm-3991322-supplementary.pdf]

Table S1. Variables associated with discordant LDL-C / apoB based on the univariate and multivariate logistic regression analysis. ASCVD – atherosclerotic cardiovascular disease; BMI – body mass index; CI – confidence interval; DM – diabetes mellitus; eGFR – estimated glomerular filtration rate; Lp(a) – lipoprotein (a); OR – odds ratio

Analyzed parameter	Univariate logistic regression			Multivariate logistic regression		
	OR	95% CI	P-value	OR	95% CI	P-value
Age, [years]	0.98	0.978-0.983	<0.001	0.986	0.983-0.99	<0.001
Male gender	0.72	0.633-0.822	<0.001	0.788	0.674-0.92	0.003
BMI, [kg/m2]	0.97	0.957-0.982	<0.001			
Smoker	1.02	0.848-1.235	0.813			
ASCVD	0.48	0.419-0.552	<0.001			
Atrial fibrillation	0.60	0.502-0.705	<0.001			
DM	0.45	0.373-0.551	<0.001	0.598	0.469-0.76	<0.001
Arterial hypertension	0.58	0.504-0.666	<0.001			
eGFR, [ml/min]	1.01	1.008-1.012	<0.001			
Aortic stenosis	0.50	0.401-0.620	<0.001			
Mitral valve disease	0.56	0.419-0.735	<0.001	0.731	0.567-0.94	0.015
History of pancreatitis	0.54	0.263-1.087	0.084			
Lipid-lowering treatment	0.45	0.381-0.532	<0.001	0.696	0.562-0.86	<0.001
Lp(a), [mg/dL]	0.998	0.996-1.00	0.042			

Table S2. Variables associated with discordant LDL-C / non-HDL-C based on the univariate and multivariate logistic regression analysis. ASCVD – atherosclerotic cardiovascular disease; BMI – body mass index; CI – confidence interval; DM – diabetes mellitus; eGFR – estimated glomerular filtration rate; Lp(a) – lipoprotein (a); OR – odds ratio

Analyzed parameter	Univariate log regression			Multivariate log regression		
	OR	95% CI	P-value	OR	95% CI	P-value
Age, [years]	0.981	0.977-0.984	<0.001	0.98	0.978-0.986	<0.001
Male gender	0.87	0.739-1.026	0.098			
BMI, [kg/m2]	0.96	0.948-0.980	<0.001			
Smokers	1.10	0.871-1.385	0.428			
ASCVD	0.542	0.455-0.645	<0.001			
Atrial fibrillation	0.684	0.553-0.845	<0.001			
DM	0.56	0.436-0.708	<0.001	0.67	0.493-0.908	0.01
Arterial hypertension	0.59	0.495-0.708	<0.001			
eGFR, [ml/min]	1.01	1.008-1.012	<0.001			
Aortic stenosis	0.63	0.446-0.902	0.011			
Mitral valve disease	0.56	0.420-0.733	<0.001			
History of pancreatitis	0.45	0.162-1.236	0.121			
Lipid-lowering treatment	0.60	0.485-0.729	<0.001			
Lp(a), [mg/dL]	1.001	0.999-1.003	0.523			

Table S3. Variables associated with discordant apoB / non-HDL-C based on the univariate and multivariate logistic regression analysis. ASCVD – atherosclerotic cardiovascular disease; BMI – body mass index; CI – confidence interval; DM – diabetes mellitus; eGFR – estimated glomerular filtration rate; Lp(a) – lipoprotein (a); OR – odds ratio

Analyzed parameter	Univariate log regression			Multivariate log regression		
	OR	95% CI	P-value	OR	95% CI	P-value
Age, [years]	0.988	0.985-0.992	<0.001			
Male gender	0.76	0.644-0.899	0.001			
BMI, [kg/m2]	0.985	0.969-1.00	0.056			

Smokers	1.03	0.807-1.304	0.836			
ASCVD	0.57	0.476-0.677	<0.001	0.78	0.628-0.875	0.029
Atrial fibrillation	0.58	0.464-0.729	<0.001			
DM	0.50	0.383-0.639	<0.001	0.61	0.444-0.829	0.002
Arterial hypertension	0.71	0.591-0.841	<0.001			
eGFR, [ml/min]	1.005	1.003-1.007	<0.001			
Aortic stenosis	0.48	0.319-0.710	<0.001			
Mitral valve disease	0.52	0.392-0.698	<0.001			
History of pancreatitis	1.16	0.568-2.353	0.689			
Lipid-lowering treatment	0.51	0.411-0.632	<0.001	0.68	0.516-0.903	0.007
Lp(a), [mg/dL]	0.998	0.995-1.00	0.078			
